# Focus on the impact of social factors and lifestyle on the disease burden of low back pain: findings from the global burden of disease study 2019

**DOI:** 10.1186/s12891-023-06772-5

**Published:** 2023-08-26

**Authors:** Yao Yang, Xigui Lai, Conghui Li, Yujie Yang, Shanshan Gu, Weiqian Hou, Liwen Zhai, Yi Zhu

**Affiliations:** 1 The Fifth Affiliated Hospital of Zhengzhou University, Zhengzhou University, Zhengzhou, Henan, 450000 China; 2 Academy of Medical Sciences, Zhengzhou University, Zhengzhou, Henan, 450000 China; 3 School of Exercise and Health, Shanghai University of Sport, Shanghai, 200438 China; 4 School of Rehabilitation Sciences and Engineering, University of Health and Rehabilitation Sciences, Qingdao, 266071 Shandong China; 5 Department of Physical Therapy, University of Toronto, Toronto, ON Canada

**Keywords:** Disability-adjusted life years, GBD, Low back pain, Risk factors

## Abstract

**Background:**

Low back pain (LBP) is one of the leading causes of disability worldwide. Differences in social backgrounds and lifestyles in various regions and countries may contribute to the discrepancies in the disease burden of LBP.

**Methods:**

Based on the GBD 2019, we collected and analyzed numbers and age-standardized rates (ASR) of LBP disability-adjusted life years (DALYs). Temporal trends in ASR were also analyzed using estimated annual percentage change (EAPC). The Age-period-cohort (APC) model was used to estimate age, period and cohort trends in DALYs of LBP. An autoregressive integrated moving average (ARIMA) model was used to forecast DALYs of LBP trends from 2020 to 2035.

**Results:**

The DALYs due to LBP increased from 1990 to 2019. The APC model showed that the risk of DALYs for global LBP increased with age and year and that the risk of DALYs was lower in the later-born cohort than in the earlier-born cohort. The main risk factors which GBD estimates were available for DALYs of LBP include smoking, occupational ergonomic factors and high BMI. It is expected that DALYs of LBP will continue to rise until 2035.

**Conclusion:**

From 1990 to 2019, the global disease burden of LBP remained high. It is necessary to pay attention to the influence of social factors and lifestyle on LBP. Focusing on the impact of social factors as well as lifestyle on the prognosis of LBP and targeting interventions may further reduce the disease burden of LBP.

## Introduction

Low back pain (LBP), a common musculoskeletal disorder, is considered one of the leading contributors of disability and causes heavy economic burden on the individual level and the global health system [1]. In 2019, LBP ranked the sixth cause of disability-adjusted life years (DALYs) in females and was the fourth cause of DALYs in age groups 25–46 years [2].

Patients who present with acute or persistent LBP may experience significant improvement in the first six weeks from the initial onset, however, the effects gradually slows down thereafter. Therefore, residual low to moderate pain and disability can persist to affect patients [3], hugely reducing their quality of life and increasing the time to return-to-work. Current research has focused on the “yellow flag signs”, the psychological and social aspects of the long-term disability caused by low back pain [4]. Related research focuses on the effects of emotions, pressure, and social stressors on LBP patients, as well as the early detection and treatment of LBP patients who are at higher risk of disability through screening or assessment tools for secondary prevention [5–7]. However, many factors, including timing, skills, and context, may limit the effectiveness of stratified treatment for LBP [4, 8].

Healthy lifestyle behavior such as refraining from smoking, being physically active, and weight control are among the modifiable influencers [9]. By changing these factors, LBP could be better prevented and managed [10].

DALYs are the total healthy life years lost from disease onset to the end of life. It is a comprehensive indicator to quantitatively calculate the healthy life years lost due to premature death and disability caused by various diseases. Through the study of DALYs in patients with low back pain, we can analyze the differences in disease burden among different regions and countries, understand the multiple factors affecting DALYs of low back pain, multi-dimensional prevention and treatment of low back pain, and reduce symptom-related disability.

## Methods

### Data source

The Global Burden of Disease (GBD) Study 2019 was performed by the Institute of Health Metrics and Evaluation (IHME). The global data on the DALYs burden of LBP from 1990 to 2019 were obtained from the Global Health Data Exchange GBD Results Tool (https://vizhub.healthdata.org/gbd-results/).

GBD 2019 low back pain disease burden data from the Global Health Data Exchange (GHDx). GBD2019 study was developed and coordinated by the Institute for Health Metrics and Evaluation (IHME) at the University of Washington, which provides rigorous and comparable measurements of important global health issues. GBD 2019 provides comprehensive estimates for 204 countries and territories for 369 causes of death, 87 diseases, injuries, etc. These data sources include household surveys, censuses, vital statistics, and civil registration. Each of these types of data was identified through a systematic review of published studies, searches of government and international organization websites, published reports, primary data sources such as demographic and health surveys, and contributions to the dataset by GBD collaborators. GBD 2019 complies with the Guidelines for Accurate and Transparent Health Estimates Reporting (GATHER) statement.

The Human Development Index (HDI), which measures the degree of socioeconomic development of countries, includes life expectancy at birth, years of schooling (include average and expected years of education), and per capita income. Given the correlation of LBP with multiple social variables [11], we assessed the correlation between DALYs due to LBP and the level of social development in different countries based on the 2019 HDI data extracted from the United Nations Development Programme (UNDP) database in the Human Development Report. (https://hdr.undp.org/data-center/human-development-index#/indicies/HDI)

All of the data used in the study were publicly available, so ethical approval was not required.

### Case definition

In GBD2019, the data of LBP were mainly derived from previous studies and publicly published data. IHME searched for relevant studies published from October 2016 to October 2017 in PUBMED, Ovid Medline, EMBase and CINAHL electronic databases. There were no age, sex, or language restrictions. A comprehensive literature search was performed using the subject words “backache”, “lumbago”, “lumbar pain,“, “back pain”, in combination with: “prevalence”, “incidence”, “cross-sectional” and “epidemiology”. Exclusion criteria included studies that were not representative of the national population, studies that were not population-based, studies with small sample size (less than 150), and studies that were not original. Additional information was obtained from the survey unit record data of the Global Health Data Exchange (GHDx), population health data, including the World Health Survey and the National Health Survey. In addition, claims data from 2000, 2010–2012, and 2014–2016 for USA states were included.

Low back pain is defined as a local pain (with or without pain referred into one or both lower limbs) that lasts for at least one day. The low back, also known as the lumbar region, is the area on the posterior aspect of the body from the lower margin of the twelfth ribs to the lower gluteal folds.

ICD-10 codes for LBP are M54.3, M54.4 and M54.5. The ICD-9 code is 724^2^.

### Statistical analysis

This study uses age-standardized rates (ASR) to measure the estimated annual percentage change (EAPC) in DALY rates to quantify the global burden of LBP. The standardized rate is measured in units per 100,000 people, and the ASR trend provides a useful description of how the disease is changing in the population. Thus, we can use ASR to develop more targeted prevention and treatment strategies. EAPC is the natural logarithm of the regression line compliance ratio, y = α + βx + ε, where y = ln (ASR) and x=calendar year. ε is the error, and EAPC is reported with a 95% confidence interval (CI). EAPC is the trend in ASR over a specific time interval. When the EAPC estimate is > 0, the ASR is considered to be on an upward trend. Conversely, when the EAPC estimate is < 0, the ASR is considered to be in a downward trend. Otherwise, ASR was considered stable over time. The estimated annual percentage change from 1990 to 2019 calculated by a linear regression model reflects the trends in the age-standardized DALYs rate of LBP disease burden.

We used an age-period-cohort (APC) model to develop estimates of the independent effects of age, period, and birth cohort on the burden of disease in LBP. Model results show longitudinal age profiles, cycle relative risk, and cohort relative risk. The age-period-cohort model is based on a Poisson distribution and is often used in epidemiological analyses of disease. The APC model captures trends in LBP DALYs by age, period, and cohort, respectively, as well as trends in relative risk at different time points.

Age refers to the age of the study subject at the time of data collection, and increasing age may be accompanied by changes in physiological characteristics as well as changes in social influences. Period refers to the year in which the data were collected, and the social environment and medical technology may differ between periods. The cohort refers to the birth cohort in which the subject was born, and subjects in different birth cohorts may have been exposed to different factors.

In the age-period-cohort model, the intrinsic estimator (IE) was used to estimate the effect coefficients of age, period and cohort effects independently. The Akaike information criterion (AIC), Bayesian information criterion (BIC), bias and log-likelihood ratio were used to evaluate the degree of fit. The relative risk (RR) of the incidence can be obtained by the natural log transformation of the effect coefficient, RR = E$xp$ (B). The age, the calendar period, and the birth cohort were all set to intervals of 5 years.

The age-period-cohort model was conducted by Stata 18.0 IE software package, and the effect coefficient was used to describe the global risk level of low back pain and its changes. R software was used for visual analysis.

The HDI is a composite measure of health, education and income. The HDI ranges from 0 to 1, with higher values indicating higher levels of socioeconomic development. Association of age-standardized DALYs rate with HDI were tested via Pearson correlation and Linear regression analyses.

The autoregressive integrated moving average (ARIMA) model is one of the most widely used models in time series analysis [12, 13]. The first step is to identify whether the data is a smooth series or not, and to smooth the non-smooth series. The corresponding model is built based on the calculated values. And test whether the model is statistically significant. The prediction results are evaluated according to the fitting effect of the model and the prediction effect. p > 0.05 shows that the model has a good fit effect using the Ljung-box test. We predicted the number of disability-adjusted life years (DALYs) attributable to LBP from 2020 to 2035 by the ARIMA model.

All data collation and analysis were performed by R (version 4.2.1) software.

## Results

### The DALYs trend of LBP at the global level

**Fig. 1:**
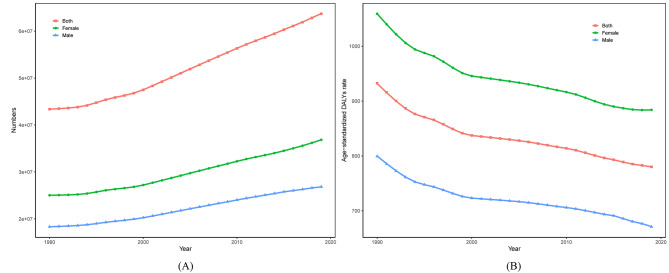
**(A)** Trend of DALYs number of LBP in the global from 1990 to 2019. **(B)** Trend of Age-standardized DALYs rate of LBP in the global from 1990 to 2019

Abbreviations: *DALYs*: Disability-Adjusted Life Years.

The number of DALYs due to low back pain increased from 1990 to 2019, higher in females than males (Fig. 1A), whereas the age-standardized DALYs rate were generally declining (Fig. 1B). In 2019, low back pain caused 63685119.8 (95% UI 44999198.1-85192922.2) cases of DALYs worldwide, which increased by 46.87% compared with 43361648.4 (95% UI 30529531.7-57934972.7) numbers of DALYs in 1990. The global age-standardized DALYs rate of LBP in 2019 was 780.2 per 100,000 population (95% UI 549.3-1046.1), decreased by 16.33% compared with the rate of 932.5 per 100,000 population (95% UI 658.6-1248.3) in 1990, a decrease of approximately 0.51% (95% CI -0.56 to -0.46) per year.

### The DALYs trend of LBP at the GBD regions level

At the level of regions, from the socio-demographic index (SDI) side, in 2019, high SDI regions have the highest age-standardized DALYs rate at 1142.7 (95% UI 809.8-1522.5) per 100,000 people, while low and medium SDI regions have the lowest at 676.7 (95% UI 479.1-903.2) per 100,000 people. Looking at the 21 GBD regions, Eastern Europe had the highest age-standardized DALYs rate of 1032.1 (95% UI 728.1-1374.1) per 100,000, as compared to East Asia which had the lowest age-standardized DALYs rate of 589.1 (95% UI 418.5-790.7) per 100,000.

From 1990 to 2019, the low-middle SDI area was the fast decrease in age-standardized DALYs rate (EAPC=-0.55, 95% UI -0.63–0.46), and the low SDI area was the slowest (EAPC=-0.26, 95%UI -0.3–0.22). At the regional level, the age-standardized DALYs rate showed a downward trend in most regions, with the most drastic decline in South Asia, followed by East Asia (Table 1).

**Fig. 2:**
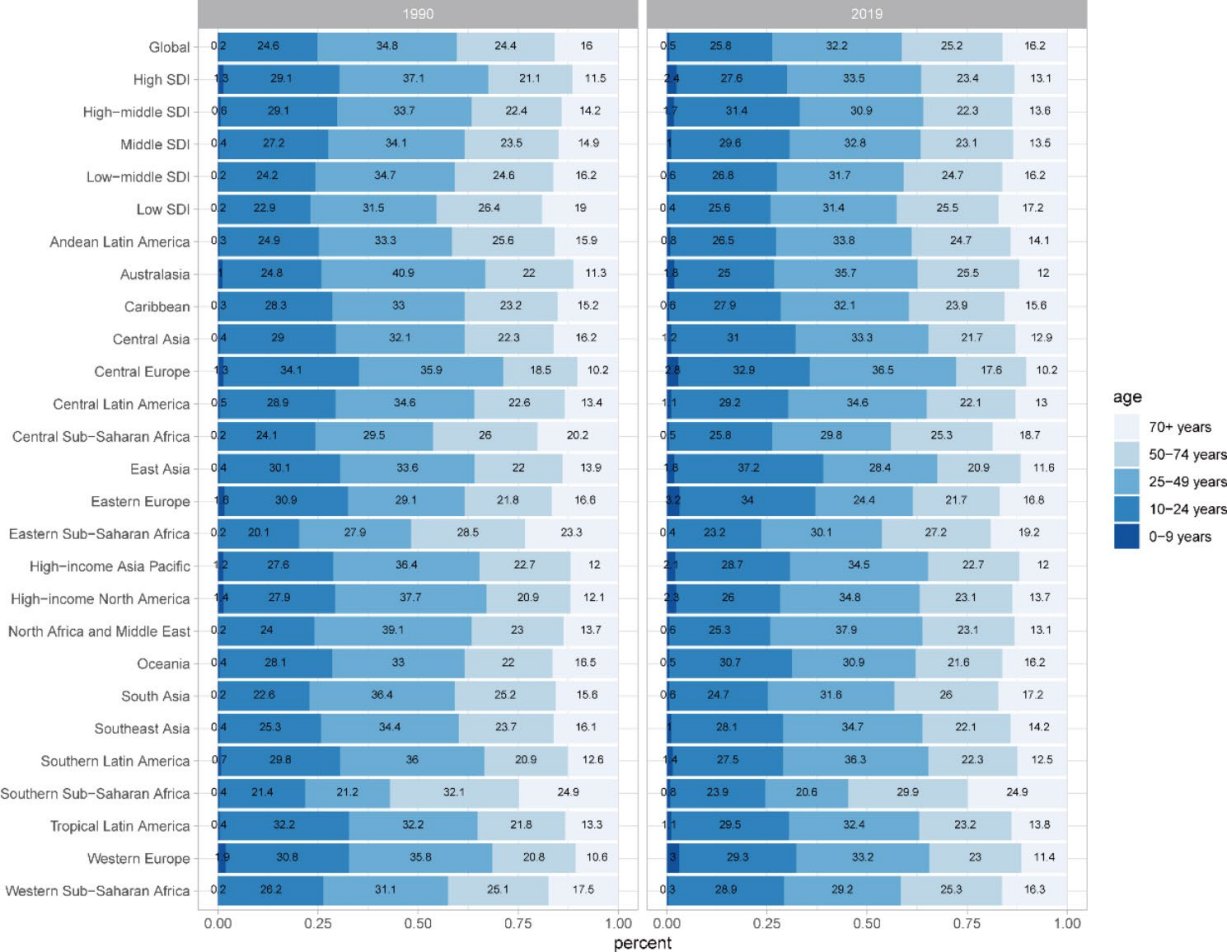
Proportion of DALYs due to LBP grouped by region and age groups in 1990 and 2019

There is no significant difference in the age distribution of DALYs for LBP in 1990 and 2019. Compared with 1990, the proportion of DALYs with LBP increased slightly in 2019 among adolescents younger than 24 years and middle-aged and older adults older than 50 years globally. People aged 10–50 years accounted for the largest proportion of DALYs with LBP (Fig. 2). Proportion of DALYs was highest in the 25–49 age group globally in 2019, but it was declined compared to 1990. The combined proportion of DALYs in the 10–24 age group and the 25–49 age group exceeded 50%. In terms of GBD regions, the proportion of DALYs due to LBP were highest in the 25–49 age group in all regions except East Asia, Eastern Europe, and Southern Sub-Saharan Africa.

**Table 1:** The DALYs cases and ASR of LBP in 1990 and 2019, as well as EAPC and 95% CI

location	1990		2019		
	DALYs cases	ASR	DALYs cases	ASR	EAPC and 95%CI
Global	43361648.4 (30529531.7-57934972.7)	932.5 (658.6-1248.3)	63685119.8 (44999198.1-85192922.2)	780.2 (549.3-1046.1)	-0.51 (-0.56–0.46)
High SDI	11866371.2 (8403598.9-15900094.8)	1277.3 (901.9–1722)	15,069,735 (10794608.4-20202296.4)	1142.7 (809.8-1522.5)	-0.28 (-0.31–0.25)
High-middle SDI	10578040.8 (7455311.6-14153811.3)	928.9 (656.2-1240.4)	13773460.2 (9743929.8-18598942.7)	773.9 (546.3-1038.2)	-0.51 (-0.57–0.45)
Middle SDI	11704806.8 (8232174.5-15511252.2)	844.3 (594-1124.6)	18452124.7 (12995697.3-24705516)	714.1 (504.6-953.5)	-0.4 (-0.48–0.31)
Low-middle SDI	6591458.7 (4659611.5-8783458.6)	795.1 (562.6-1058.6)	10876183.3 (7675136.9-14492257.5)	676.7 (479.1-903.2)	-0.55 (-0.63–0.46)
Low SDI	2599842.2 (1833596.8-3473087.2)	758 (536.8-1011.2)	5476316.2 (3848252.6-7290440.4)	707.1 (499.3-942.6)	-0.26 (-0.3–0.22)
Andean Latin America	196527.7 (137947.7-263814)	676.6 (477.5-908.6)	415525.3 (293398-559273.7)	674.4 (476.3-909.8)	0 (-0.02-0.02)
Australasia	244516.1 (172506.3-325393.9)	1106.7 (777.3-1474.4)	353808.1 (248870.4-476776.7)	988.3 (687.2-1338.3)	-0.34 (-0.41–0.27)
Caribbean	229719.7 (161919.1-306269.7)	741.4 (523.5-986.1)	364602.3 (257466.1-485551.5)	727.5 (513.2–965)	-0.07 (-0.09–0.05)
Central Asia	469577.1 (331256.5-628993.3)	839.2 (591.8-1125.3)	731,962 (513799.1-980790.8)	827.8 (582.9-1105.7)	-0.07 (-0.07–0.06)
Central Europe	1570425.3 (1104359.1-2103370)	1154.4 (810.1-1541.1)	1709097.2 (1209435.2-2309586.3)	1127 (788.5-1509.6)	-0.12 (-0.13–0.11)
Central Latin America	976052.5 (679355.2-1307709.5)	766.5 (538-1021.9)	1953805.1 (1372397.7-2601136)	768.6 (541.1-1024.7)	-0.09 (-0.12–0.06)
Central Sub-Saharan Africa	260115.6 (183372.6-346068.3)	747.9 (527-1003.3)	644392.4 (451536.8-864330.2)	745.7 (524.6-1000.1)	-0.03 (-0.05–0.01)
East Asia	8945835.2 (6299975.1-11927210.1)	822.5 (580.1-1103.9)	10885998.9 (7718845.4-14745714.4)	589.1 (418.5-790.7)	-0.69 (-0.88–0.5)
Eastern Europe	2,685,570 (1900734.3-3601516.8)	1050.8 (746.4-1403.4)	2847346.8 (2024234.8-3815425)	1032.1 (728.1-1374.1)	-0.03 (-0.05–0.01)
Eastern Sub-Saharan Africa	807,659 (567706.2-1066150.8)	716.7 (506-957.7)	1869808.9 (1303043.6-2481458.9)	714.2 (501.8-957.4)	-0.01 (-0.03-0)
High-income Asia Pacific	2419392.9 (1686880.5-3268379.5)	1228 (856.6-1656.2)	2884797.5 (2038411.4-3897904.8)	1080.5 (751-1464.9)	-0.28 (-0.33–0.23)
High-income North America	4794243.5 (3387557.2-6425706.4)	1537.3 (1085.9-2059.4)	6,174,175 (4449895.5-8124406.1)	1362.1 (975.7-1800.2)	-0.27 (-0.33–0.21)
North Africa and Middle East	2,274,265 (1590071.7-3033869.8)	916.6 (648.6-1222.1)	4899300.2 (3404484.7-6533346.7)	862 (605.5-1153.3)	-0.18 (-0.2–0.17)
Oceania	45345.2 (31871.8-60396)	985.3 (697.7-1300.8)	102524.5 (72075.3-136527.2)	983.6 (695.2-1303.9)	0.01 (-0.01-0.03)
South Asia	6224377.6 (4389237.1-8313649.3)	773.5 (548.5-1027.2)	9920979.5 (7027196.4-13254436.6)	603.5 (427-809.8)	-0.91 (-1.06–0.76)
Southeast Asia	3289256.6 (2324619.6-4351603.7)	914.1 (646.6-1215.4)	6174959.4 (4329885.7-8252199.4)	900 (634.6-1203.1)	-0.06 (-0.1–0.03)
Southern Latin America	381,925 (264387.3-517643.9)	800.4 (555.3-1082.1)	590175.8 (411523.4-800737.9)	790.3 (550.1–1073)	0 (-0.02-0.02)
Southern Sub-Saharan Africa	251168.8 (177392.9-332823.7)	676.9 (477.9-903.3)	442883.4 (312871.9-592296.2)	638.8 (452.3-853.4)	-0.13 (-0.16–0.1)
Tropical Latin America	1091784.3 (768328.6-1463865.7)	847.5 (598.5-1127.4)	2098383.5 (1480053.8-2786301.1)	860.3 (606.3-1146.1)	-0.05 (-0.08–0.03)
Western Europe	5195116.1 (3673879.6-6950463.2)	1133.6 (798.3-1520.9)	6174812.7 (4342038.8-8320107.5)	1062.6 (745.6-1441.7)	-0.21 (-0.23–0.19)
Western Sub-Saharan Africa	1008775.3 (711663-1352599.9)	792.1 (560-1058.1)	2445781.3 (1718562-3281522.6)	795.7 (560.4-1065.4)	-0.02 (-0.05-0.01)

### The DALYs trend of LBP in the nations

A total of 204 countries and territories were included in GBD 2019. The top 10 countries with the largest increase in the number of DALYs have all increased more than 200% from 1990 to 2019. Qatar had the largest increase in the number of DALYs, with an elevation of nearly 691%. The United Arab Emirates came in second (Table 2).

**Table 2:** The top 10 countries with the largest percentage change in DALYs cases of LBP between 1990 and 2019

Nations	Cases in 1990	Cases in 2019		Percentage of change in cases (%)	
Qatar	3248		25,695		691
United Arab Emirates	12,221		89,026		628
Jordan	19,963		81,779		310
Bahrain	3363		13,742		309
Maldives	1146		3846		236
Kuwait	11,860		39,639		234
Djibouti	1918		6383		233
Equatorial Guinea	2039		6686		228
Saudi Arabia	89,128		291,699		227
Oman	11,049		35,426		221

### Age-period-cohort model analysis of global DALYs rate

**Fig. 3:**
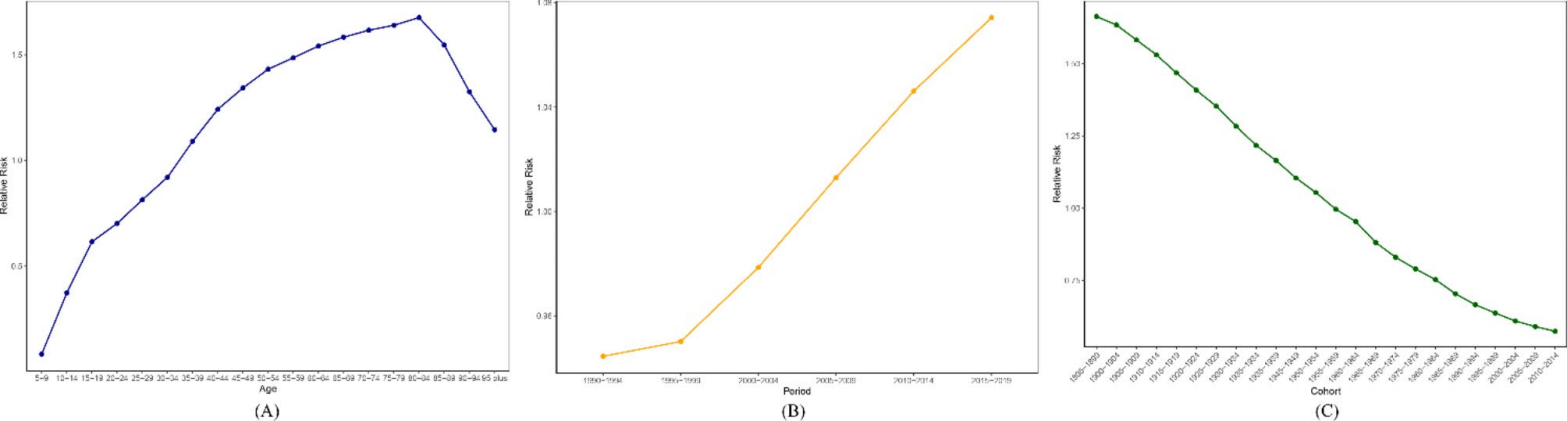
Age-period cohort effects of LBP globally (1990–2019). **(A)** Age effect **(B)** Period effect **(C)** Cohort effect

After excluding the effects of cycle and cohort, we found a correlation between LBP DALYs rate and age. The global risk of DALYs rate for LBP increased with age, reaching a maximum at 80–84 years. For patients with LBP, their DALYs rate risk was 19.96 times higher at 80–84 years than at 5–9 years, a smaller peak in DALYs rate risk was observed for patients with LBP at 15–19 years, and patients with LBP at 85 years and older DALYs rate risk started to decrease (Fig. 3A). As the period effect shows, the period RR in global DALYs rate displayed an obvious upward trend, especially after 1990–2000 (Fig. 3B). The cohort effect of the LBP DALYs rate decreased with the increase of the birth year worldwide (Fig. 3C).

### Factors affecting the DALYs of LBP in global and regions

**Fig. 4:**
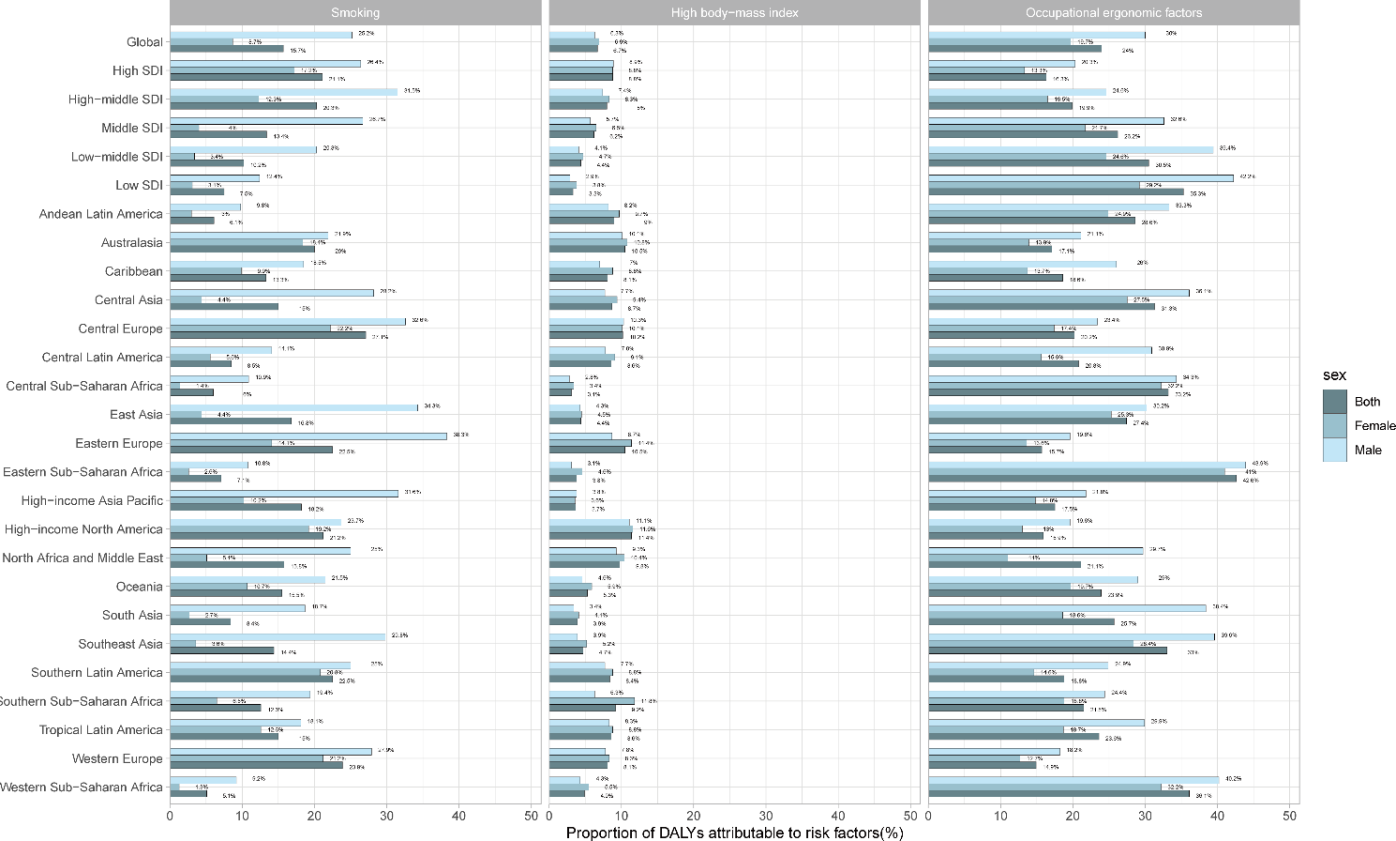
Proportions of low back pain DALYs attributable to different risk factors and grouped by sex in 2019

There are three main risk factors influencing the DALYs of LBP worldwide according to GBD estimates, 15.7% (95% UI 11.8–19.6) attributable to smoking, 6.7% (3.9–9.8) attributable to high BMI, and 24% (22.4–25.6) attributable to occupational ergonomic factors. The proportions of DALYs in both high SDI and high − middle SDI regions are mainly attributed to smoking, while occupational ergonomic factors is the primary risk factor in other SDI regions. Besides, the impact of these risk factors varies by region. Central Europe is the region with the highest proportion attributable to smoking (27.1%), while the highest percentages of high body-mass index and occupational ergonomic factors are found in High-income North America (11.4%) and Eastern Sub-Saharan Africa (42.6%), respectively. Globally from a gender perspective, DALYs of LBP attributable to smoking and occupational ergonomic factors were higher in men, while DALYs of LBP attributable to high BMI was higher in women. In high SDI areas, the main factor contributing to LBP DALYs in both men and women was smoking. For men, smoking was the major factor for LBP DALYs in high-middle SDI areas, while for women, it was occupational ergonomic factors. Occupational ergonomic factors are the main factor contributing to the DALYs of LBP in both men and women in other SDI areas (Fig. 4).

**Fig. 5:**
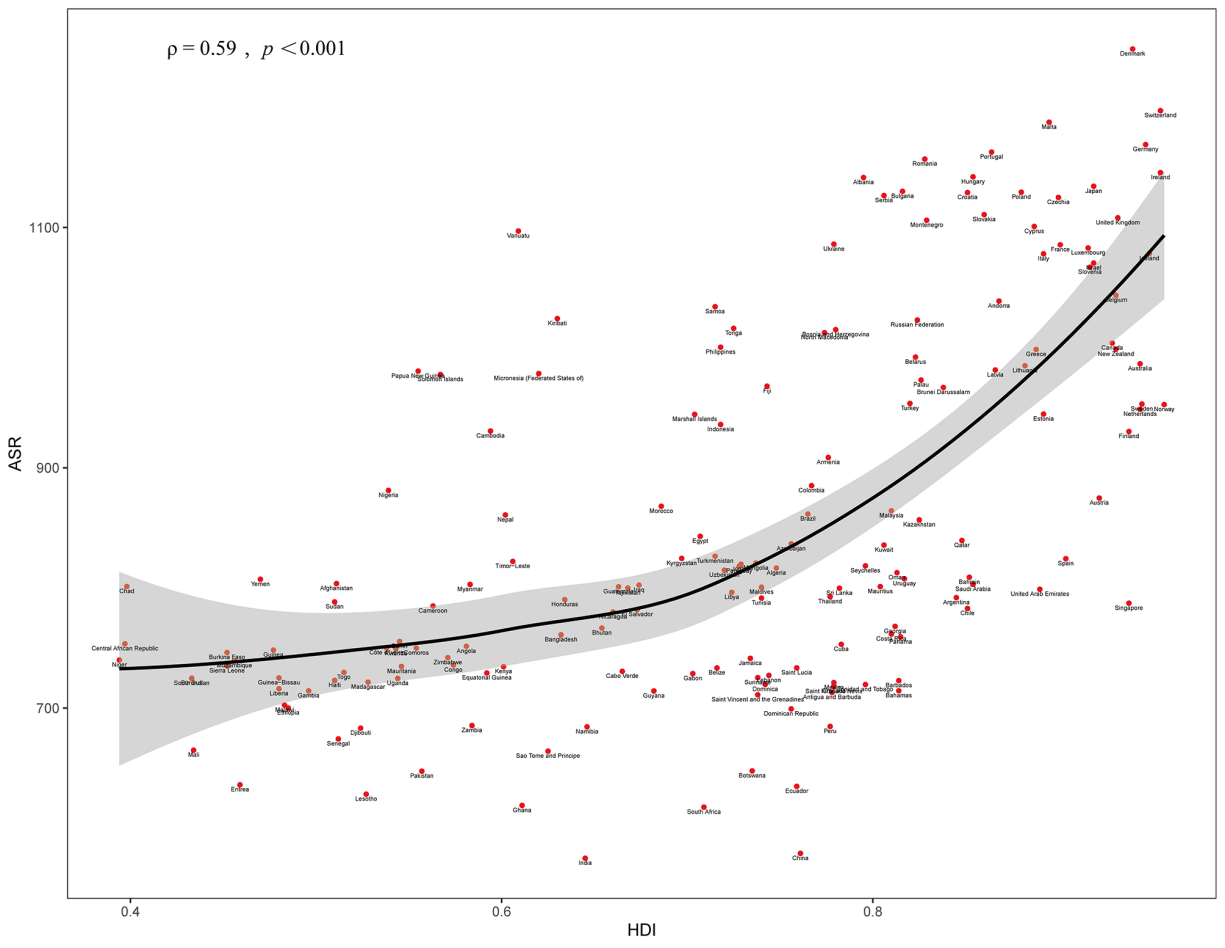
Correlation analysis between the HDI and LBP age-standardized DALYs rate in 2019. The dots represent countries with available HDI data. The ρ indices and p values were derived from Pearson correlation analysis

In 2019, HDI was positively correlated with age-standardized rates of DALYs (Fig. 5), meaning that countries with high HDI may also have higher rates of standardized DALYs. This indicates that even in countries with higher per capita income, life expectancy, and years of education, there may still be a higher burden of LBP.

### DALYs projections for low back pain in 2020–2035

**Fig. 6:**
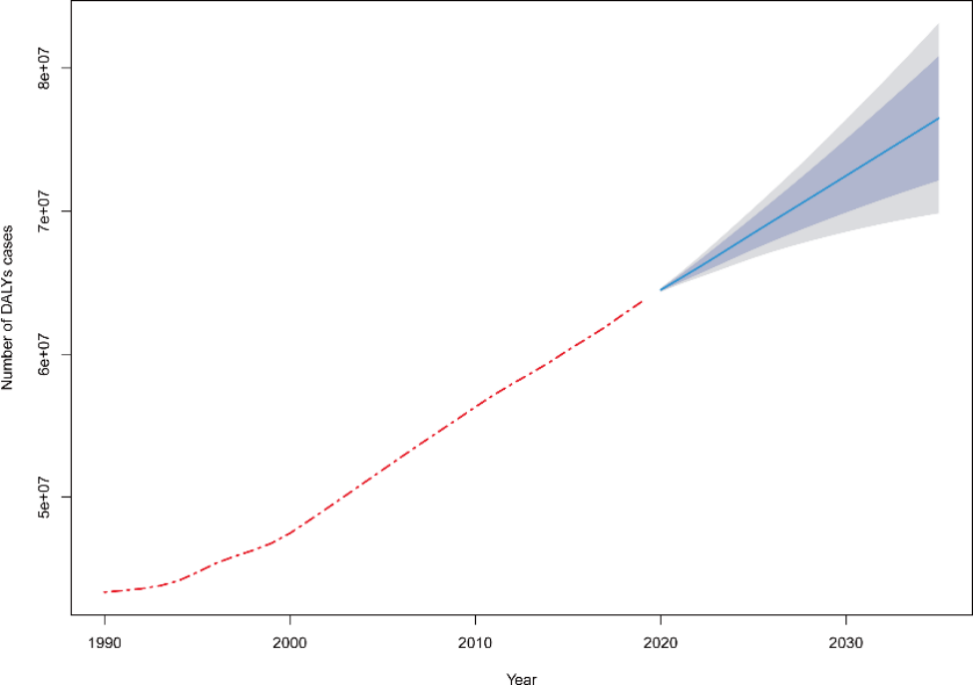
Forecast of DALYs numbers with LBP from 2020–2035 through ARIMA. The blue line indicates the predicted value, the blue area indicates the 95%CI of the predicted value, and the gray is the 80%CI

ARIMA time series model was used to predict the number of DALYs with LBP. The results showed that ARIMA (2,2,0) was an appropriate model to predict the global number of DALYs trend with LBP. Ljung-box test indicates that the model fitted well. From 2020 to 2035,the number of DALYs for LBP is still increasing globally(Fig. 6).

## Discussion

### Global distribution of DALYs for low back pain

Low back pain is one of the leading causes of disability worldwide [14]. From 1990 to 2019, the global DALYs caused by low back pain had increased. Despite the progress of medical advancements and the growing global population, the burden of LBP continues to grow. However, age-standardized DALYs rates have been declining. Nevertheless, the downward trend has gradually moderated in recent years.

Any age group can suffer from low back pain. As age increases, the risk of disability due to low back pain also increases. Age is one of the factors that affect the disability of low back pain, and it should be noted that the number of adolescents with low back pain has also been increasing in recent years. The period effect generally shows an upward trend with the passing years. While low back pain has been well treated with the progress in medical advancements, improved medical care system is essential to carry out the patient education and symptom management for patients with low back pain comprehensively, due to its complex etiological mechanism. The overall decreasing trend of the cohort effect with the year of birth may be due to the fact that with social progress and economic development, people’s lifestyles are changing, and health education on LBP prevention has also become increasingly popular in recent years. More people are becoming aware of the importance of physical activity, which is beneficial in reducing the risk of disability. It is expected that the number of DALYs caused by LBP will continue to increase until 2035, which will still require a lot of medical resources and costs.

### Regional differences in the distribution of DALYs for low back pain

The level of economic development and social progress have a significant impact on the DALYs of individuals suffering from LBP. In all SDI score areas, the EAPC values were negative, indicating that the age-standardized rate of DALYs decreased from 1990 to 2019. This suggests that educational attainment and income can help reduce DALYs associated with LBP. Although the rate of age-labeled DALYs for low back pain in high SDI areas showed a decreasing trend, it was less pronounced compared to low SDI areas. Interestingly, high-income areas also appeared to have higher rates of age-standardized DALYs, indicating that the influencing factors may vary by region. Higher-income regions may also face a higher burden of low back pain despite having higher level of social development.

### National differences in the distribution of DALYs for low back pain

From 1990 to 2019, six out of the top ten countries with the highest growth in the number of low back pain DALYs were Gulf Cooperation Council (GCC) countries. In fact, Qatar and the United Arab Emirates had a staggering growth rate of more than 600% in the number of low back pain DALYs. The GCC countries have witnessed a surge in obesity, which has become a major and growing health problem due to rising incomes, rapid urbanization and improved living conditions [15–18]. This has led to sedentary and less physically active lifestyle habits due to various factors such as gender, cultural background and geographical conditions, possibly resulting in elevated DALYs in low back pain [19]. Insufficient physical activity is a risk factor for many diseases, and previous studies have shown that the prevalence of insufficient physical activity may be higher in high-income countries than in low-income countries [20].

The correlation analysis of HDI with ASR has confirmed that higher quality of life may be accompanied by a higher disease burden of LBP. As the quality of life improves, the ASR also increased, suggesting that the quality of life and disease burden are interrelated. Research indicates that patients with low back pain are negatively affected by factors such as low income [21], lower social class [22], and years of education [23]. However, our study highlights that countries with high per capita income and quality of life may still experience a significant burden of LBP. Some developed countries may also be at risk of a high disease burden of LBP, and that certain modifiable lifestyles can contribute to this issue.

Interestingly, both Equatorial Guinea and Djibouti fall under the Sub-Saharan Africa (SSA), and previous studies have found that the prevalence and risk factors for chronic LBP in SSA are similar to those in high-income countries. However, due to differences in socioeconomic and healthcare systems, low-and-middle-income countries may face a more significant burden of disease [24].

It is important to recognize that the outcomes of patients with chronic LBP can vary based on their educational level as well as socioeconomic status. Acknowledging the impact of social factors on the disease process of LBP can help reduce the disease burden of LBP and provide useful insights for improving LBP treatment strategies.

### Differences in demographic and occupational characteristics should guide preventive and treatment measures

Low back pain can arise from a combination of factors and manifests itself in various physical or psychological conditions [25]. Hence, it is crucial to provide suitable healthcare education and treatment based on different demographic features and occupations.

The number of DALYs attributed to LBP is higher in women than men, as well as the age-standardized rate. LBP is the most common musculoskeletal condition experienced by women during pregnancy [26]. Additionally, women who initially encounter low back pain during pregnancy may continue to experience persistent pain for up to a year postpartum [27], which may significantly affect their daily life and work, especially in severe cases [28]. Our findings are consistent with earlier studies that report the association between LBP and smoking in males, and LBP and occupational ergonomic factors in females [29].

Occupations that require moderate-to-vigorous physical activity or prolonged sedentary period may increase the likelihood of developing LBP [27]. Suffering from persisting LBP may result in functional impairment in patients, affecting their quality of life and work productivity. The loss of productivity and increased sick leave caused by low back pain can further increase the economic burden on society [30, 31]. Even after appropriate treatment, patients with low back pain may still exhibit poor prognosis [32]. Interventions aimed to target various occupations at the workplace maybe necessary to formulate appropriate measures [33–35].

The risk of DALYs of LBP tends to increases with age but there is a smaller peak among individuals aged 15–19 years. Given that this is a critical developmental stage for adolescents undergoing growth, several factors such as body posture, gender, psychological status, and prolonged use of electronics may contribute to low back pain [36, 37]. Notably, adolescent low back pain can also serve as a predictor of persistent low back pain in adulthood, underscoring the importance of targeted interventions to minimize disability risk [38, 39].

With an aging population and a significant increase in middle-aged workers engaged in physically demanding jobs for extended periods, there is a higher likelihood of developing long-term absenteeism and disability due to LBP [39]. Additionally, higher level of occupational physical activity in midlife was significantly associated with worse activities of daily living in late life [40]. Hence, appropriate treatment and occupational-related interventions for middle-aged individuals experiencing LBP can help reduce the risk of disability in the elderly population.

### Focus on social factors and modifiable lifestyles for patients with low back pain

Physically demanding work, smoking, and working conditions are often overlooked factors in preventing and managing LBP. Modifiable lifestyles such as smoking and obesity are predictors of pain and dysfunction related to LBP [41, 42]. By guiding lifestyle changes in people with LBP, it may be possible to reduce the risk of long-term pain and disability. Moreover, studies show that occupational therapy and lifestyle interventions are feasible treatments for chronic pain in adults [43]. Occupational physiotherapy through external interventions in conjunction with different regional and national health policies may be able to further reduce DALYs for LBP.

Biopsychosocial interventions for low back pain are significant, especially at the social level. Although current interventions based on the biopsychosocial medicine model have been proven to be effective, their impact on reducing work-related disability remains unclear [44–46]. Insufficient compensation benefit systems, medical facilities, and family support may hinder patients’ ability to return to work and society [47–49]. A “system-wide” intervention that considers various work demands and social support could effectively reduce DALYs resulting from low back pain, taking into account the national context [50]. Over the next 15 years, DALYs associated with low back pain are projected to continue rising, necessitating continuous effort to improve the system centered on low back pain to minimize the risk of disability.

### Limitation

There are a few limitations in this study. Firstly, the differences in disease diagnostic criteria in from year to year inevitably lead to certain deviations in the results when GBD collects data, and these data are not directly measured. The completeness of data obtained through mathematical models may also have an impact on the results, and these deviations have been reported in other articles [1]. Secondly, due to a lack of relevant information, DALYs for individual LBP may be underestimated, and the quality and completeness of reported data may be inconsistent across different countries. In addition, the etiology of LBP is complex, and the risk factors we examined are limited to those provided in the GBD database, which may not be comprehensive. Despite these limitations, our study contributes a new perspective to the management of LBP disorders.

## Conclusions

The disease burden of LBP may still be increasing and the risk of disability associated with LBP tends to rise with age. Being a musculoskeletal disorder, low back pain is influenced by various factors, including smoking, occupational behaviors and high BMI. Furthermore, the socio-economic status and gender of different countries also impact DALYs related to LBP. Analyzing the trends of DALYs associated with LBP while considering the socio-cultural backgrounds and lifestyles of different regions and countries could aid relevant health authorities and policy decision makers in formulating and modifying public health policies. A multidisciplinary approach, an active and healthy lifestyle, and highlighting the role of occupational therapy in LBP management may be effective measures to minimize the disease burden of LBP.

## Data Availability

The datasets generated and/or analysed during the current study are available in the Global Burden of Disease repository, http://ghdx.healthdata.org/gbd-results-tool.

## Abbreviations

ARIMA: Autoregressive integrated moving average
ASR: Age-standardized rates
CI: Confidence interval
DALYs: Disability-adjusted life years
EAPC: Estimated annual percentage change
GBD: Global Burden of Disease
HDI: Human Development Index
LBP: Low back pain
SDI: Socio-demographic Index
UI: Uncertainty interva
